# Pulmonary infection caused by *Schizophyllum commune*: a case study

**DOI:** 10.3389/fmed.2025.1475896

**Published:** 2025-06-16

**Authors:** Chunxue Xue, Jiangtao Shen, Jie Song, Jingjing Yang, Shuming Zhang, Jinxiang Wang

**Affiliations:** ^1^Department of Respiratory and Critical Care Medicine, Beijing Luhe Hospital, Capital Medical University, Beijing, China; ^2^Department of Orthopedics, Beijing Luhe Hospital, Capital Medical University, Beijing, China

**Keywords:** pulmonary infection, pulmonary mycosis, *Schizophyllum commune*, *S. commune*, allergic bronchopulmonary mycosis

## Abstract

Although pulmonary infections caused by *Schizophyllum commune* (*S. commune*) are still relatively rare in clinical practice, they are increasingly familiar and valued by clinicians. The clinical symptoms and imaging of pulmonary infection with *S. commune* are not typical, thus diagnosis needs to be confirmed by clinical etiology. *S. commune* is one of the main fungal species causing allergic bronchopulmonary mycosis. The overall prognosis of *S. commune* pulmonary infection is good. This paper describes a case of a woman with no underlying disease who had pulmonary infection with *S. commune*. This report aims to improve clinicians’ awareness of the disease.

## Introduction

*Schizophyllum commune* (*S. commune*), which belongs to the Phylum Basidiomycota, order Agaricales, Family Schizophyllaceae, and genus *Schizophyllum*, is a conditional pathogenic fungus. While *S. commune* is ubiquitously distributed in nature, predominantly colonizing decaying wood and plant debris, its role in human disease remains uncommon. Globally, reported cases exhibit distinct geographical clustering, with the majority documented in Japan and other Asian countries, likely attributable to heightened clinical awareness and advanced diagnostic practices in these regions (1–3). Sporadic cases have also been reported in Europe, North America, and India, though at substantially lower frequencies (4, 5). Pulmonary *S. commune* infections are a rare form pulmonary mycosis. For instance, Chowdhary et al. (6) analyzed clinical data of 143 cases of ABPM infected by fungi other than aspergillus, and found that the commonest etiologic agent was *Candida albicans* (60%), followed by *Bipolaris species* (13%), *S. commune* (11%). This low incidence may reflect both diagnostic challenges — due to its morphological resemblance to contaminant fungi and the requirement for specialized culture techniques — and potential underreporting in resource-limited settings. In this paper, we summarize the clinical manifestations, imaging characteristics, bronchoscopic manifestations and therapeutic procedure of pulmonary *S. commune* infection, with a view to improve clinicians’ level of understanding.

## Materials and methods

### Conventional testing

Conventional testing included bacterial, mycobacterial, and fungal culture and smears. Furthermore, the detection of 1,3-β-glucan and galactomannan was conducted respectively for *Candida* and *Aspergillus*.

### Metagenomic next-generation sequencing (mNGS) assay

Following standard procedures (7), bronchoalveolar lavage fluid (BALF) specimens were collected by experienced physicians and utilized for mNGS analysis. BALF samples were sent to WillingMed Technology (Beijing) Co., Ltd, for mNGS analysis. Nucleic acid extraction and purification, library construction and quantitative analysis, high-throughput sequencing and bioinformatics data analysis were performed and pathogen reports were generated according to standard procedures (8, 9).

### Hematoxylin and Eosin (H&E) staining

Tissue sections (4 μm) were deparaffinized in xylene, rehydrated through graded ethanol series, and stained with Harris hematoxylin (5 min). After differentiation in 1% acid ethanol and bluing in 0.2% ammonia water, sections were counterstained with eosin Y (1 min), dehydrated, cleared in xylene, and mounted with resinous medium.

### Grocott’s Methenamine Silver (GMS) staining

Paraffin-embedded necrotic tissue sections (5 μm) were dewaxed, rehydrated, and oxidized with 1% periodic acid (10 min). After borax pretreatment (1%, 5 min), sections were stained with preheated hexamethylenetetramine-silver nitrate (60°C, 30–60 min) until fungal hyphae displayed black metallic deposits. Unbound silver was removed with 2% sodium thiosulfate (2 min), followed by eosin counterstaining (0.1%, 30 sec). Slides were dehydrated and mounted.

## Case report

**FIGURE 1:**
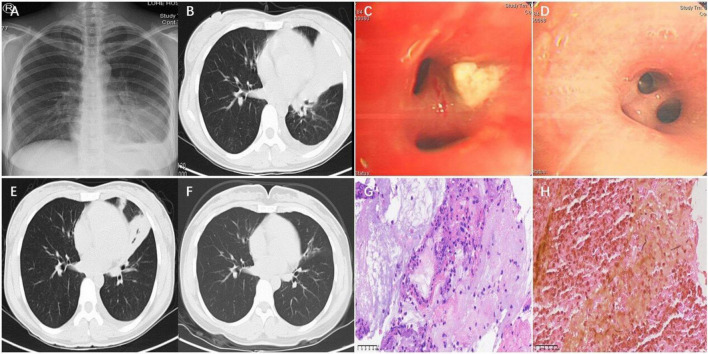
Chest X-ray showed left lower lung patch with pleural effusion **(A)**. Chest CT showed large blurred consolidation shadows in the left lung and crescent-shaped liquid density areas in the left thoracic cavity **(B)**. Bronchoscopy of the opening in the left lingual lobe revealed a white necrotic material that blocked the lumen on January 21 **(C)**. Repeat bronchoscopy showed that there were no yellow secretions obstructing the left lingual lobe on January 26 **(D)**. Re-examination of the chest CT revealed less consolidation of the left lung and left pleural effusion than before on January 29 **(E)**. Chest CT showed mild bronchiectasis in the left lingual lobe on April 24 **(F)**. Histopathological examination (H&E staining, magnification, **×**400) **(G)**. Grocott’s Methenamine Silver Staining, magnification, **×**400 **(H)**.

A 30-year-old female patient was admitted at our department of respiratory and critical care medicine on January 19, 2022, with cough for more than half a month and fever for 2 weeks. Her main symptoms were cough and a small amount of yellow phlegm accompanied by fever. She also presented with headache as well as chest and back pain. After her symptoms failed to improve following empirical antibiotic therapy (cefazolin and azithromycin), she developed intermittent fever and hemoptysis. She had no prior underlying medical conditions. This patient was a yoga instructor, and used a humidifier for a long time before onset of the aforementioned symptoms. Initial chest x-ray (CXR) revealed left lower lung patch with pleural effusion (Figure 1A). After admission, she was initially diagnosed with community-acquired pneumonia. She was intravenously given moxifloxacin, but still manifested recurrent fever and hemoptysis. The allergen-specific IgE testing of her peripheral blood revealed a total serum IgE level of 2226.00 KU/L, with allergen-specific concentrations as follows: *Aspergillus fumigatus* (0.62 IU/ml), *Dermatophagoides pteronyssinus* (2.9 IU/ml), and a combined fungal panel (*Candida albicans*/*Penicillium notatum*/*Alternaria alternata*/*Cladosporium herbarum*/*Aspergillus niger*) at 5.0 IU/ml. These values were graded as 1.7, 2.7, and 3.1, respectively, indicating a progressively increasing trend in allergen-specific sensitization levels. Peripheral blood eosinophils account for 10%. The dynamic erythrocyte sedimentation rate was 98 mm/h, while tests for galactomannan and (1, 3)-β-D glucan revealed negative results. Carcinoembryonic antigen was 10.64 U/L, while the fractional exhaled nitric oxide was 42 ppb. A computed tomography (CT) scan of the chest revealed multiple patchy blurred shadows in both lungs, the left of which showed large blurred consolidation shadows, and crescent-shaped liquid density areas in the left thoracic cavity (Figure 1B). Bronchoscopy of the opening in the left lingual lobe revealed a white necrotic material that blocked the lumen. The obstructing material was removed using biopsy forceps, which resulted in a small amount of bleeding and overflow of purulent secretions (Figure 1C). Histopathological Examination staining of the necrotic material revealed inflammatory infiltration predominantly composed of lymphocytes, plasma cells, and eosinophils. The stroma exhibited interstitial edema with focal areas of degeneration and necrosis (Figure 1G). The histopathological examination of necrotic material using GMS staining revealed scattered fungal hyphae (Figure 1H). Metagenomic next-generation sequencing (mNGS) of BALF identified 547 reads of *S. commune* and 4 reads of human herpesvirus 7 (HHV-7), with the remaining sequences classified as human microbiota. The proportion of eosinophils detected in the BALF was 32%. The treatment plan was subsequently adjusted to voriconazole 200 mg q12h, administered via intravenous infusion, according to the etiology. The patient’s clinical symptoms improved significantly. Repeat bronchoscopy showed that there were no yellow secretions obstructing the left lingual lobe, although purulent secretions could still be aspirated (Figure 1D). Moreover, repeat mNGS of BALF detected no *S. commune* but only 4 reads of HHV-7, with the remaining sequences classified as human microbiota. Re-examination of the chest, via CT scan, revealed less consolidation of the left lung and left pleural effusion than before (Figure 1E). Follow-up chest CT showed mild bronchiectasis in the right middle lobe and left lingual lobe after 3 months of treatment using voriconazole (Figure 1F).

## Discussion

*S. commune* is a basidiomycete bracket fungus found commonly in rotting wood, dry logs and fallen trees. Despite its worldwide distribution, this fungus was not considered a human pathogen until 1950 when Kligman isolated it from a case of onychomycosis (10). *S. commune* was first discovered in Japan in 1994, where it was associated with allergic bronchopulmonary mycosis (ABPM) (1). The associated mycotic infections mainly targets the respiratory tract, owing to the fact that the pathogen releases basidiospores into the atmosphere which are subsequently inhaled (2). Respiratory infections from *S. Commune* mainly manifest as ABPM by mucoid impaction (3), although in rare cases they may also present as asthma (11), pulmonary fungal ball (5), honeycomb lung (12) and chronic eosinophilic pneumonia (13). It not yet clear which people are susceptible to *S. commune*, although people without previous underlying diseases and immunodeficiencies can still be affected. Based on previous cases (2), consider vigilance against this fungal infection when the population has a history of exposure to rotting trees or wild fungi. The patient in the present case had normal immune function with no underlying diseases, although it is unclear whether her low body weight and use of a humidifier before disease onset were risk factors. While immunocompetent individuals are rarely affected, prolonged humidifier use may aerosolize environmental fungi, and low body weight could reflect nutritional deficits impacting mucosal immunity—both hypotheses requiring further study. A critical limitation of this etiological investigation lies in the absence of microbial analysis of the patient’s humidifier, which precludes definitive identification of this potential environmental reservoir as the source of fungal exposure. Pulmonary infection caused by *S. commune* clinically presents with cough, followed by expectoration, and wheezing. Previous physical examinations have revealed that a small number of cases may show no clinical symptoms (4, 12, 14, 15). The patient in the present case had a subacute onset, with cough, sputum, and fever as the main symptoms, and hemoptysis.

Previous studies have shown that *S. commune* is one of the main causes of ABPM (6). When pulmonary *S. commune* infection manifests as ABPM, there is need to distinguish it from ABPA due to their similar clinical manifestations, chest imaging features and bronchoscopy findings. There is currently no standard treatment for pulmonary *S. commune* infection, with the present clinical treatment modalities mainly involving drug and physical therapy. The commonly used drugs include antifungals, glucocorticoids and expectorants. There is a challenge on which type of antifungal drugs to be selected, and the length of the course of treatment. At the moment, no standard modality exists for treatment of this condition. *In vitro* susceptibility studies demonstrate that itraconazole, voriconazole, posaconazole, and amphotericin B exhibit potent activity against *S. commune* (2). Clinical case reports indicate that itraconazole and voriconazole are the most commonly prescribed agents. While prednisone monotherapy has been reported to achieve lesion resolution in isolated cases (13), the absence of long-term follow-up data precludes definitive conclusions regarding its efficacy in eradicating *S. commune.* Furthermore, although inhaled corticosteroids (e.g., budesonide) may transiently alleviate symptoms, mycological clearance is not achieved, as evidenced by persistent positive cultures in treated patients (11). Physical therapy mainly includes bronchoscopy to remove phlegm plugs (16) and surgical resection of the lesions. Additional research evidence has shown that the overall prognosis of patients with pulmonary *S. commune* infection is good, with only one death (which was related to occurrence of serious complications and the patient’s resistance to voriconazole) reported so far (17). Reports have also shown that effective anti-infective treatment improves symptoms within 1–6 months, in most cases, the lesions may be completely absorbed, and some lesions will leave bronchiectasis (18, 19). We did not detect *S. commune* in the second BALF, and her condition improved after voriconazole treatment, showing bronchiectasis on imaging. While conventional clinical diagnosis for etiological identification primarily relies on microbial culture combined with microscopic examination, the absence of fungal colony isolation from the BALF specimen in the present case represents a notable diagnostic limitation. Pulmonary *S. commune* infection is a rare pulmonary fungal disease, and the clinical understanding of the disease is still lacking. Therefore, there is a need to actively improve bronchoscopy and etiological examination. The findings in the present case provides valuable insights on etiological detection of *S. commune*, and are expected to guide clinicians during diagnosis and treatment.

## Data Availability

The raw data supporting the conclusions of this article will be made available by the authors, without undue reservation.
